# Cerebral Cystic Echinococcosis

**DOI:** 10.1155/2020/1754231

**Published:** 2020-02-29

**Authors:** Abolghasem Siyadatpanah, Enrico Brunetti, Amir Emami Zeydi, Yousef Dadi Moghadam, Nelson Iván Agudelo Higuita

**Affiliations:** ^1^Ferdows School of Paramedical and Health, Birjand University of Medical Sciences, Birjand, Iran; ^2^University of Pavia, Unit of Infectious and Tropical Diseases, IRCCS San Matteo Hospital Foundation, Pavia, Italy; ^3^Department of Medical-Surgical Nursing, Nasibeh School of Nursing and Midwifery, Mazandaran University of Medical Sciences, Sari, Iran; ^4^Student Research Committee, Faculty of Medicine, Mazandaran University of Medical Sciences, Sari, Iran; ^5^Section of Infectious Diseases, Department of Internal Medicine, University of Oklahoma Health Sciences Center, Oklahoma City, USA

## Abstract

Cystic echinococcosis (CE) is a neglected helminthic disease and major public health problem in several regions of the world. The zoonosis is caused by the larval stage of different cestode species belonging to the genus *Echinococcus.* CE can affect any organ with the liver and lungs being most commonly involved. The brain is involved in less than 2% of the cases. We report a case of a CE1 echinococcal cyst of the brain in an Iranian patient.

## 1. Introduction

Cystic echinococcosis (CE) is caused by the larval stage of different cestode species of the genus *Echinococcus.* The zoonosis represents a formidable public health challenge given its global distribution, neglected status, and problems with the implementation of control programs [1–4]. Approximately 58% of the total population of Central Asian countries (i.e., Iran, Kazakhstan, Tajikistan, Turkmenistan, Uzbekistan, Afghanistan, Mongolia, Pakistan, and Western China) is at risk of contracting the disease [5]. In Iran, CE is one of the most important parasitic diseases with a prevalence rate estimated to be between 1.18–3 per 100,000 population [6]. CE costs Iran more than US$230 million per year equating to approximately 0.03% of the country's Gross Domestic Product [7].

Most patients have a single cystic lesion located in a single organ with the liver being affected most often, but the presentation can be highly variable [1, 8, 9]. Cystic echinococcosis restricted to the brain is a rare entity representing only 1-2% of the cases and occurring most commonly in children [10, 11]. Some authors have proposed that the combination of a patent ductus arteriosus and ingestion of unboiled animals' milk might explain the preponderance of pediatric cases [12]. We report a case of cerebral CE in an adult from Iran.

## 2. Case Report

A 39-year-old male was referred to the Khatamolanbia Hospital from the city of Gonbad-e Kavus in the province of Golestan with a two-week history of headache.

**Figure 1:**
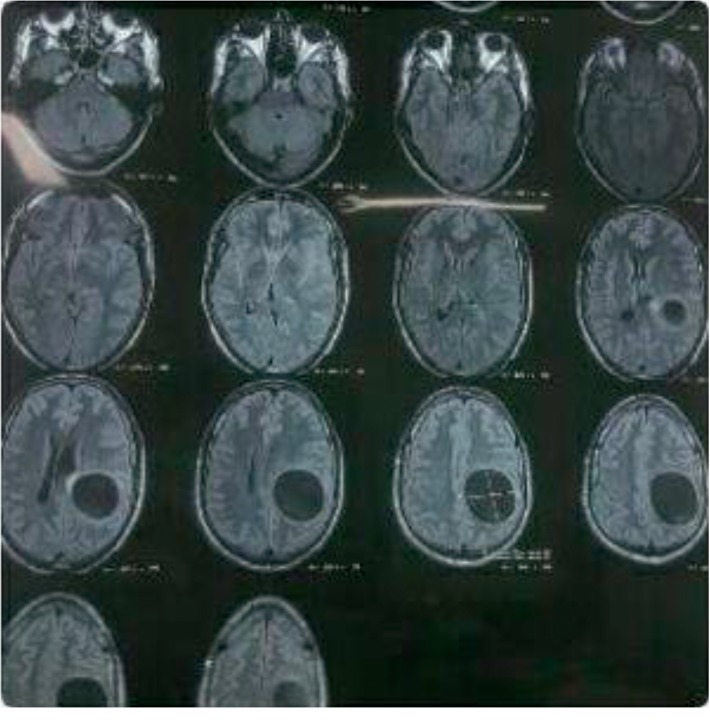
CT scan showing a large cyst affecting the left fronto-parieto-occipital region.

**Figure 2:**
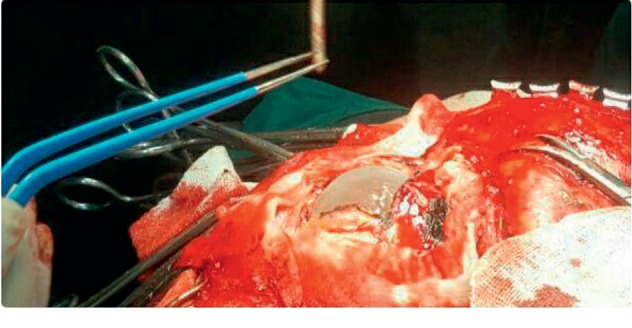
A large cyst is evident after resection of the skull and opening of the dura.

**Figure 3:**
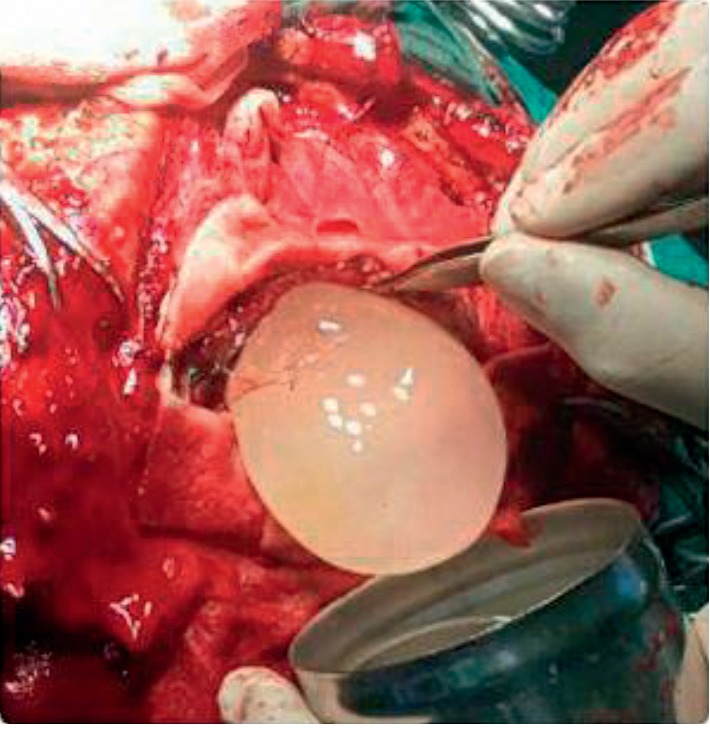
Removal of the cyst using the Dowling-Orlando technique with aid of gravity.

**Figure 4:**
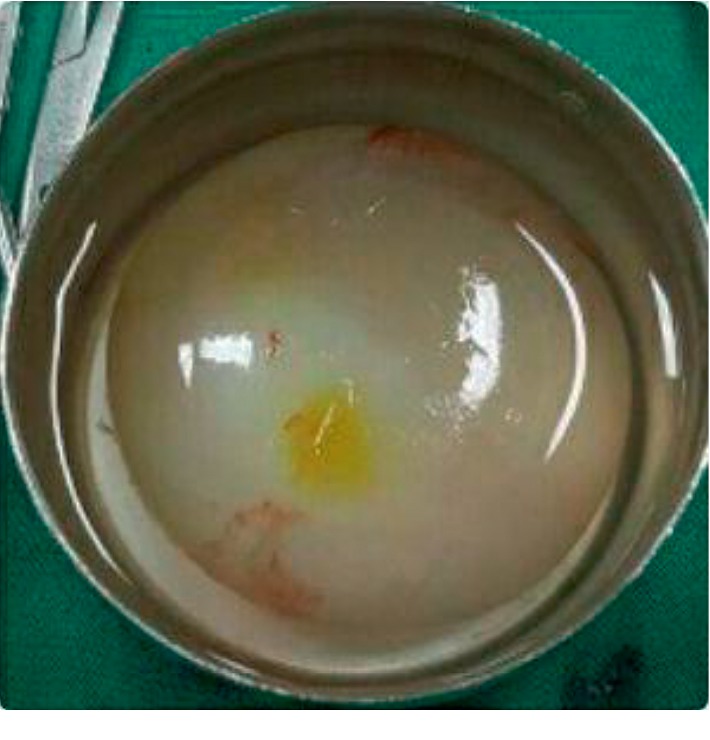
Intact hydatid cyst completely removed without complications.

**Figure 5:**
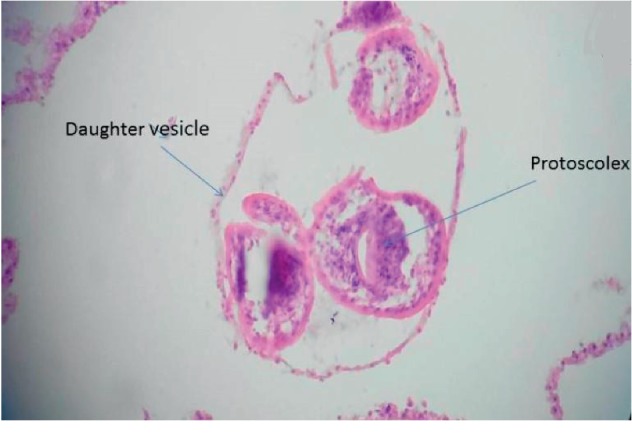
Hematoxylin and eosin stain of formalin-fixed paraffin-embedded cell block of cyst fluid (magnification, ×40).

The patient worked in animal husbandry and kept a dog to protect the flock. Routine laboratory tests on admission showed a white blood cell (WBC) count of 5.5 × 10^9^/L, normal eosinophil count, hemoglobin, and biochemical parameters. The patient was discharged and returned one month later with right hemisensory loss, severe headache, and loss of balance. A magnetic resonance imaging (MRI) of the brain showed a large cyst located on the left fronto-parieto-occipital region without surrounding edema, compressing the lateral ventricles and causing shift of the midline structures (Figure 1). An ultrasound of the liver and spleen showed no cysts. The patient was taken to surgery, and the cyst was removed using the Dowling-Orlando technique with the aid of gravity without rupture (Figures 2–4). Histopathological examination with hematoxylin and eosin staining confirmed the diagnosis of cystic echinococcosis (Figure 5).

The patient was treated with albendazole at a dose of 15 mg/kg daily for four weeks postoperatively with no side effects. The patient is doing well approximately 14 months after discharge from the hospital.

## 3. Discussion

Cystic echinococcosis is a complex disease with many factors contributing to its morbidity and mortality; it is seen in the most impoverished areas of the planet. The cohabitation of humans and domestic animals is vital to the persistence of the disease in many regions of the world [13–16]. The development and outcome of infection is dependent on the parasite's avoidance of immune response strategies and the host's immunity [17].

*E. granulosus* comprises 10 genotypes (G1 to G10) which have been assigned to different species. *E. granulosus* sensu stricto (G1, G2, and G3), *E. equinus* (G4), *E. ortleppi* (G5), and *E. canadensis* (G6, G7, G8, G9, and G10) [18, 19]. All genotypes have the ability to cause disease with *E. granulosus* sensu stricto (G1, G2, and G3) being responsible for the bulk of cases worldwide [20, 21]. There is also suggestion that tissue tropism is in part determined by the genotype. For example, the cervid genotype (G8) most frequently localizes in the lung, and genotype 6 (G6) might have a higher affinity for the brain. Human infection by genotype 4 has not been described [22–24]. Genotyping was unfortunately not performed in this case due to financial limitations.

The signs and symptoms of cerebral CE are those of a space occupying lesion, i.e., headache, nausea, vomiting, and papilledema. Focal neurological findings such as motor or sensory deficits, seizures, visual impairment, and gait and speech disturbances are also common [10, 25, 26]. Lesions are usually solitary and the location of the cyst(s) in the brain varies; most tend to be supratentorial and localize in the watershed area of the middle cerebral artery [10, 25]. It is important to evaluate for extraneural involvement with abdominal and cardiac imaging in cases of cerebral CE, as this scenario is not uncommon [11, 26, 27].

As with other anatomical locations, the diagnosis is suspected based on imaging. Computerized tomography (CT) and magnetic resonance imaging (MRI) are the modalities most commonly used to support diagnosis and therapeutic planning. Cerebral cystic echinococcosis in both imaging techniques frequently presents in the CE1 stage as a unilocular, smooth, thin-walled fluid filled spherical cystic lesion with the fluid of the same density as the cerebrospinal fluid [27]. MRI usually shows hypointense lesions on T1-weighted images and hyperintense lesions on T2-weighted images. The cyst wall can have a rim of low signal intensity in both T1- and T2-weighted images. Perilesional edema or enhancement is usually absent unless the cyst is superinfected [27]. Magnetic Resonance Spectroscopy (MRS) can be used *in vivo* not just to diagnose the parasitic nature of the cyst in the brain but also, given that different cyst stages have different metabolic profiles [28], to monitor response to treatment beyond standard imaging [29].

The differential diagnosis of cerebral CE in the CE1 stage includes arachnoid cysts, porencephaly, cystic astrocytoma, cystic primary and metastatic tumors, brain abscess, coenurosis, and neurocysticercosis [25, 27].

All cyst stages have been shown to occur in the brain [30] although the concept of a stage-specific approach that should guide choice of treatment in the liver has limited application in the brain. Surgery is the treatment of choice with Dowling's technique being commonly used for the evacuation of the cyst [11, 31, 32]. Preoperative determination of the location and number of cysts is of paramount importance. Postoperative complications are not uncommon. The intraoperative rupture rates vary between 17% and 26%. Other common immediate postoperative complications include subdural fluid collections and hydrocephalus. Visual loss and seizures were the most frequent long-term complications from a large case series in Turkey [11, 32].

The efficacy and safety of scolicidal agents such as hypertonic saline have not been prospectively studied although hypertonic saline has been used when intraoperative spillage occurs [11, 26, 32]. Medical treatment with albendazole at a dose of 10–15 mg/kg daily has been recommended when there is rupture or puncture of the hydatid cyst, recurrence, multiple and disseminated lesions, for preoperative cyst volume reduction, prophylactic use, and poor surgical candidacy. The length of postoperative treatment is empirical and needs to be adapted to the individual case [11, 32]. At least a few patients have been treated exclusively with albendazole but follow-up data are lacking [29, 33].

Short or inexistent follow-up in case series and the differences in surgical interventions limit the risk assessment of recurrence after puncture or rupture of a cyst but a high risk of recurrence and mortality has been reported [11, 26, 32]. The overall morbidity and mortality rates for intracranial hydatid cysts vary between 5%–10% and 4%–10%, respectively [32].

Clinicians should include CE in the differential diagnosis of focal lesions of the brain in patients coming from or who have traveled to endemic areas, keeping in mind that all cyst stages are possible in the brain exactly as in the liver, although a stage-specific approach is limited by surgery being the best treatment. Use of albendazole in cysts that cannot be treated surgically should be encouraged and reported to make experience available to the medical community.

## References

[1] Agudelo Higuita N. I., Brunetti E., McCloskey C. (2016). Cystic echinococcosis. *Journal of Clinical Microbiology*.

[2] Otero-Abad B., Torgerson P. R. (2013). A systematic review of the epidemiology of echinococcosis in domestic and wild animals. *PLoS Neglected Tropical Diseases*.

[3] Brunetti E., Garcia H. H., Junghanss T. (2011). Cystic echinococcosis: chronic, complex, and still neglected. *PLoS Neglected Tropical Diseases*.

[4] Craig P. S., McManus D. P., Lightowlers M. W. (2007). Prevention and control of cystic echinococcosis. *The Lancet Infectious Diseases*.

[5] Zhang W., Zhang Z., Wu W. (2015). Epidemiology and control of echinococcosis in central Asia, with particular reference to the People’s Republic of China. *Acta Tropica*.

[6] Rokni M. B. (2008). The present status of human helminthic diseases in Iran. *Annals of Tropical Medicine & Parasitology*.

[7] Fasihi Harandi M., Budke C. M., Rostami S. (2012). The monetary burden of cystic echinococcosis in Iran. *PLoS Neglected Tropical Diseases*.

[8] Belli S., Akbulut S., Erbay G., Kocer N. E. (2014). Spontaneous giant splenic hydatid cyst rupture causing fatal anaphylactic shock: a case report and brief literature review. *The Turkish Journal of Gastroenterology*.

[9] Engin G., Acunaş B., Rozanes İ., Acunaş G. (2000). Hydatid disease with unusual localization. *European Radiology*.

[10] Ersahin Y., Mutluer S., Güzelbag E. (1993). Intracranial hydatid cysts in children. *Neurosurgery*.

[11] Ciurea A. V., Fountas K. N., Coman T. C. (2006). Long-term surgical outcome in patients with intracranial hydatid cyst. *Acta Neurochirurgica*.

[12] Lunardi P., Missori P., Di Lorenzo N., Fortuna A. (1991). Cerebral hydatidosis in childhood. *Neurosurgery*.

[13] Carmena D., Sánchez-Serrano L. P., Barbero-Martínez I. (2008). *Echinococcus* granulosus infection in Spain. *Zoonoses and Public Health*.

[14] Buishi I., Njoroge E., Zeyhle E., Rogan M. T., Craig P. S. (2006). Canine echinococcosis in Turkana (north-western Kenya): a coproantigen survey in the previous hydatid-control area and an analysis of risk factors. *Annals of Tropical Medicine & Parasitology*.

[15] Buishi I., Walters T., Guildea Z., Craig P., Palmer S. (2005). Reemergence of canine *Echinococcus* granulosus infection, wales. *Emerging Infectious Diseases*.

[16] Buishi I. E., Njoroge E. M., Bouamra O., Craig P. S. (2005). Canine echinococcosis in northwest Libya: assessment of coproantigen ELISA, and a survey of infection with analysis of risk-factors. *Veterinary Parasitology*.

[17] Siracusano A., Delunardo F., Teggi A., Ortona E. (2012). Host-parasite relationship in cystic echinococcosis: an evolving story. *Clinical and Developmental Immunology*.

[18] McManus D. P. (2013). Current status of the genetics and molecular taxonomy of *Echinococcus*species. *Parasitology*.

[19] Nakao M., Lavikainen A., Yanagida T., Ito A. (2013). Phylogenetic systematics of the genus *Echinococcus* (Cestoda: Taeniidae). *International Journal for Parasitology*.

[20] Cucher M. A., Macchiaroli N., Baldi G. (2016). Cystic echinococcosis in South America: systematic review of species and genotypes of *Echinococcus* granulosus sensu latoin humans and natural domestic hosts. *Tropical Medicine & International Health*.

[21] Ito A., Nakao M., Lavikainen A., Hoberg E. (2017). Cystic echinococcosis: Future perspectives of molecular epidemiology. *Acta Tropica*.

[22] Moro P., Schantz P. M. (2006). Cystic Echinococcosis in the Americas. *Parasitology International*.

[23] Sadjjadi S. M., Mikaeili F., Karamian M. (2013). Evidence that the *Echinococcus* granulosus G6 genotype has an affinity for the brain in humans. *International Journal for Parasitology*.

[24] Alvarez Rojas C. A., Romig T., Lightowlers M. W. (2014). *Echinococcus* granulosus sensu lato genotypes infecting humans—review of current knowledge. *International Journal for Parasitology*.

[25] Gezen F., Baysefer A., Koksel T., Gonul E., Melih Akay K., Erdogan E. (1995). Hydatid cysts of the brain. *Clinical Infectious Diseases*.

[26] Turgut M. (2001). Intracranial hydatidosis in Turkey: its clinical presentation, diagnostic studies, surgical management, and outcome. A review of 276 cases. *Neurosurgical Review*.

[27] Tüzün M., Altınörs N., Arda İ. S., Hekimoğlu B. (2002). Cerebral hydatid disease. *Clinical Imaging*.

[28] Hosch W., Junghanss T., Stojkovic M. (2008). Metabolic viability assessment of cystic echinococcosis using high-field1H MRS of cyst contents. *NMR in Biomedicine*.

[29] Seckin H., Yagmurlu B., Yigitkanli K., Kars H. Z. (2008). Metabolic changes during successful medical therapy for brain hydatid cyst: case report. *Surgical Neurology*.

[30] Razek A. A. K. A., El-Shamam O., Wahab N. A. (2009). Magnetic resonance appearance of cerebral cystic echinococcosis: world health organization (WHO) classification. *Acta Radiologica*.

[31] Carrea R., Dowling E., Guevara J. A. (1975). Surgical treatment of hydatid cysts of the central nervous system in the pediatric age (Dowling’s technique). *Pediatric Neurosurgery*.

[32] Altinörs N., Bavbek M., Caner H. H., Erdoğan B. (2000). Central nervous system hydatidosis in Turkey: a cooperative study and literature survey analysis of 458 cases. *Journal of Neurosurgery*.

[33] Svrckova P., Nabarro L., Chiodini P. L., Jäger H. R. (2019). Disseminated cerebral hydatid disease (multiple intracranial echinococcosis). *Practical Neurology*.

